# Enhanced DHA Production in *Aurantiochytrium* by ω-3 Desaturase Integration and Fatty Acid Synthase Disruption

**DOI:** 10.3390/md24040144

**Published:** 2026-04-20

**Authors:** Ziyu Wang, Yujian Wang, Weijian Wan, Chao Chen, Wen Wen, Xiaojin Song, Jinsong Xuan, Yingang Feng

**Affiliations:** 1 Department of Bioscience and Bioengineering, School of Chemistry and Biological Engineering, University of Science and Technology Beijing, Beijing 100083, China; 2 CAS Key Laboratory of Biofuels, Shandong Provincial Key Laboratory of Energy Genetics, Shandong Engineering Research Center of Single Cell Oil, Qingdao Engineering Laboratory of Single Cell Oil, Qingdao Institute of Bioenergy and Bioprocess Technology, Chinese Academy of Sciences, Qingdao 266101, China; 3 Shandong Energy Institute, Qingdao 266101, China; 4 Qingdao New Energy Shandong Laboratory, Qingdao 266101, China; 5 University of Chinese Academy of Sciences, Beijing 100049, China

**Keywords:** *Aurantiochytrium*, *Schizochytrium*, ω-3 desaturase, docosahexaenoic acid, docosapentaenoic acid, metabolic engineering, polyketide synthase, polyunsaturated fatty acid, polyunsaturated fatty acid synthase

## Abstract

Docosahexaenoic acid (DHA) is an essential ω-3 polyunsaturated fatty acid (PUFA) with high nutritional and pharmaceutical value. The marine protist *Aurantiochytrium* is a promising industrial DHA producer; however, its DHA biosynthesis via the PUFA synthase pathway co-produces ω-6 docosapentaenoic acid (DPA), limiting DHA purity. Here, we introduced an ω-3 desaturase from *Phytophthora infestans* (Pin-O3D) into *Aurantiochytrium* sp. SD116. Functional validation in an *Escherichia coli* system co-expressing the native PUFA synthase confirmed that Pin-O3D converts DPA to DHA, shifting the DHA/DPA ratio from 1:1 to 2:1. *Pin-O3D* was then integrated into the fatty acid synthase (FAS) locus, simultaneously attenuating FAS activity and enabling heterologous gene expression. The engineered strain ΔFAS-Pin-O3D exhibited significantly (*p* < 0.0001 in *t*-test) increased DHA content (55.2% of total fatty acids) and DHA/DPA ratio (5.91) in shake flasks, with no negative impact on biomass or lipid accumulation. Fed-batch fermentation confirmed the scalability of this strategy, achieving a >20% increase in DHA/DPA ratio. This study demonstrates that combining heterologous ω-3 desaturase expression with FAS attenuation is an effective approach for optimizing PUFA profiles in *Aurantiochytrium*.

## 1. Introduction

Polyunsaturated fatty acids (PUFAs), especially docosahexaenoic acid (DHA, C22:6 ω-3) and eicosapentaenoic acid (EPA, C20:5 ω-3), are essential for human health, playing critical roles in brain development, cardiovascular health, and overall well-being [1,2]. The growing global demand for these beneficial fatty acids, combined with the declining availability of traditional fish oil sources due to overfishing and environmental pollution, underscores the urgent need for alternative and sustainable production platforms [3,4]. Among microbial DHA producers, thraustochytrids—including *Schizochytrium*, *Aurantiochytrium* (also known as *Schizochytrium* [5]), *Hondaea*, and *Thraustochytrium*—are regarded as the most promising industrial chassis because of their rapid growth, high lipid accumulation capacity, and remarkable DHA productivity [6,7]. Several *Schizochytrium* and *Aurantiochytrium* strains have obtained safety certifications from the US FDA and the EU EFSA and are now widely used for producing food-grade DHA [8].

*Aurantiochytrium* sp. SD116 has been extensively studied as an industrially relevant strain with robust fermentation performance and high lipid yield [9,10,11,12,13]. Unlike plants and mammals, which synthesize long-chain polyunsaturated fatty acids (LC-PUFAs) through the conventional desaturase/elongase pathway, thraustochytrids primarily produce LC-PUFAs via a polyketide synthase (PKS)-like PUFA synthase system [14,15]. The fatty acid profile of *Aurantiochytrium* sp. SD116 is relatively simple, with DHA and docosapentaenoic acid (DPA, C22:5 ω-6) as the two major LC-PUFA products, accompanied mainly by saturated fatty acids such as C14:0 and C16:0, and only trace amounts of arachidonic acid (ARA) and EPA [11], whereas ω-3 DPA constitutes less than 0.5% of total fatty acids and is therefore not reported in most studies. Since ω-3 DPA is negligible in the PUFA profile of *Aurantiochytrium* sp. SD116, in the following text, DPA will refer to ω-6 DPA unless otherwise specified. Both DHA and DPA are synthesized concurrently by the PUFA synthase in *Schizochytrium* and *Aurantiochytrium*, but the mechanism determining the DPA/DHA ratio remains unclear [16]. Unlike DPAs in fish oil, which are mainly ω-3 fatty acids, DPAs produced by thraustochytrids are mainly ω-6 fatty acids [17], and their health impact and functional properties have not been well established. The DPA/DHA ratio produced by thraustochytrids is difficult to modulate by altering fermentation conditions, making the intrinsic DPA/DHA ratio a limiting factor for DHA accumulation [18,19]. Therefore, developing an efficient metabolic route to directly convert DPA into DHA represents a promising strategy to improve DHA purity and overall production.

Metabolic engineering approaches, particularly the introduction of ω-3 desaturases, offer a direct and effective means to enhance DHA production by converting ω-6 PUFAs (e.g., DPA) into ω-3 PUFAs [18,20]. ω-3 Desaturases insert a double bond at the ω-3 position of fatty acid chains, thereby converting ω-6 fatty acids to their ω-3 counterparts [20]. Various ω-3 desaturases with distinct substrate specificities have been identified from different organisms, which exhibit distinct substrate specificities [21]. Ren et al. first reported overexpression of a heterologous ω-3 desaturase from *Saprolegnia diclina* in *Schizochytrium*, achieving a 3% conversion of DPA to DHA, suggesting that the desaturase was functional but had a low efficiency [20]. Recently, Li et al. discovered that overexpression of an ω-3 desaturase from *Parietichytrium* sp. in *Schizochytrium* increased the DHA/DPA ratio by 14.6%, indicating that ω-3 desaturases from different sources may have varying effects [18]. In 2019, Shrestha et al. expressed several ω-3 desaturases in *Nicotiana benthamiana* leaves and evaluated their ability to convert various ω-6 fatty acids into ω-3 forms. They found that a specific ω-3 desaturase (O3D) from *Phytophthora infestans* (*P. infestans* O3D, hereafter Pin-O3D) could convert approximately 80% of DPA to DHA [22]. This finding provides a strong rationale for selecting Pin-O3D to enhance DHA accumulation in *Schizochytrium* and *Aurantiochytrium*.

In this study, we aimed to improve DHA production in *Aurantiochytrium* sp. SD116 by overexpressing Pin-O3D. Recognizing the competitive relationship between the fatty acid synthesis (FAS) pathway and the PKS pathway for common precursors, we also sought to strengthen DHA synthesis by weakening the FAS pathway, as our previous work showed that inactivation of one *FAS* copy significantly increases DHA content [23]. We investigated the effects of Pin-O3D expression on the fatty acid profile, particularly DPA-to-DHA conversion, and evaluated its overall contribution to DHA biosynthesis in *Aurantiochytrium* sp. SD116. This research provides insights and strategies for metabolic engineering of oleaginous microorganisms to achieve higher and more efficient DHA production.

## 2. Results and Discussion

### 2.1. Sequence and Structural Features of Pin-O3D

**Figure 1:**
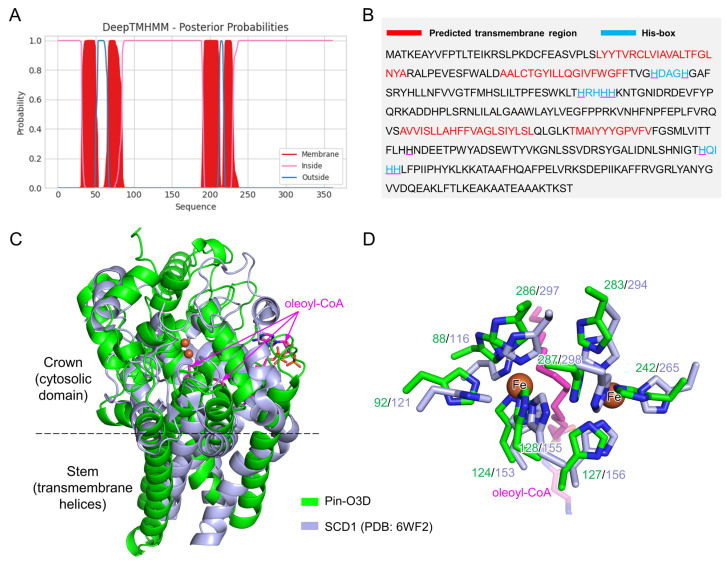
Sequence and structural analysis of Pin-O3D. (**A**) Prediction of transmembrane helices in Pin-O3D using DeepTMHMM. (**B**) The sequence of Pin-O3D. Residues in predicted transmembrane helices are colored red; histidine motifs are colored light blue; histidine residues coordinating diiron ions in the catalytic site are underlined. (**C**) Superimposition of the AlphaFold-predicted structure of Pin-O3D (green) with the reported crystal structure of mouse stearoyl-CoA desaturase 1 (SCD1, light blue). The diiron center in the SCD1 structure is shown as brown spheres, and the bound product oleoyl-CoA is depicted as sticks in magenta. (**D**) Structural details of the histidine cluster coordinating the diiron ions at the catalytic site. The nine iron-coordinating histidine residues are shown as sticks; they are all conserved in SCD1 and Pin-O3D and occupy similar spatial positions.

To assess whether Pin-O3D possesses the structural characteristics of a membrane-bound ω-3 fatty acid desaturase, sequence and structural analyses were performed. Using DeepTMHMM (https://dtu.biolib.com/DeepTMHMM, accessed on 14 November 2025) [24], Pin-O3D was predicted to be a transmembrane protein containing four helices (Figure 1A,B). Amino acid inspection revealed that Pin-O3D harbors three histidine-rich motifs (H-Box motifs 1–3) (Figure 1B), which are typical conserved features for coordinating the diiron catalytic center of membrane-bound desaturases [21]. Furthermore, analysis of the Pin-O3D structure predicted by AlphaFold (AlphaFold Database no. AF-D0NE80-F1) showed that it shares an overall mushroom-like shape, with four transmembrane helices forming the stem and the catalytic domain forming the crown (Figure 1C). Dali search results indicated that the catalytic domain is similar to that of mammalian stearoyl-CoA desaturase 1 (SCD1; PDB 6WF2) [25]. The predicted structure suggests that Pin-O3D belongs to the membrane-bound CoA-type desaturase family, with conserved histidine residues forming the catalytic site that binds diiron ions. The substrate-binding pocket is located below the diiron ions. The shape of the substrate-binding pocket and the surrounding structure vary considerably, reflecting differences in substrate selectivity between SCD1 and Pin-O3D (Figure 1D). Together, these analyses support that Pin-O3D may function as an ω-3 desaturase with potential activity toward LC-PUFAs.

### 2.2. Functional Validation of Pin-O3D in E. coli

To directly test whether Pin-O3D can catalyze the conversion of DPA (produced by the PUFA synthase) to DHA, we first established a heterologous functional validation system in *E. coli* BL21(AI) by co-expressing Pin-O3D with the PUFA synthase derived from *Aurantiochytrium* sp. SD116. The PUFA synthase consists of ORF-A, ORF-B, and ORF-C, and a phosphopantetheinyl transferase (PPTase) is required to activate the acyl-carrier protein modules [26]. In our previous work, recombinant expression of the SD116 PUFA synthase and PPTase enabled de novo biosynthesis of DHA and DPA in *E. coli* [26], providing a platform to evaluate Pin-O3D desaturation activity.

**Figure 2:**
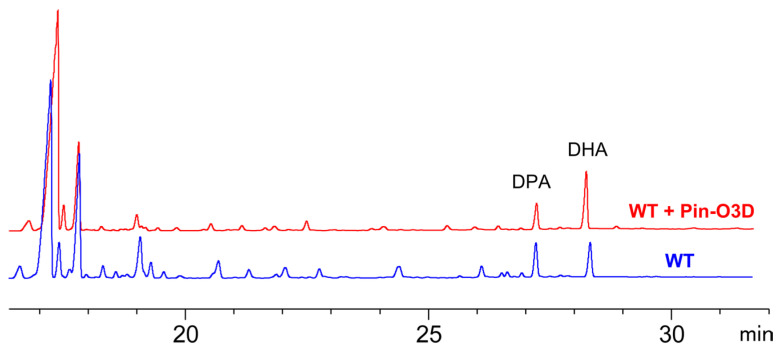
Activity analysis of Pin-O3D in *E. coli*. Gas chromatograms of total fatty acids from *E. coli* expressing the PUFA synthase (WT, blue) and co-expressing PUFA synthase and Pin-O3D (WT + Pin-O3D, red).

Total fatty acids were extracted from DHA-producing *E. coli* cells with or without Pin-O3D expression. Gas chromatography (GC) analysis showed that the strain expressing only the PUFA synthase accumulated DHA and DPA at comparable levels, resulting in an approximately 1:1 ratio (Figure 2). In contrast, introduction of Pin-O3D markedly altered the LC-PUFA profile, shifting the DHA/DPA ratio from ~1:1 to ~2:1 (Figure 2). These results provide direct functional evidence that Pin-O3D is catalytically active and can convert DPA to DHA in cooperation with the SD116 PUFA synthase. However, the DHA/DPA ratio produced by the PUFA synthase in *E. coli* differs from that in *Aurantiochytrium* sp. SD116; therefore, the effect of Pin-O3D expression needed further verification in the native host of PUFA synthase.

### 2.3. Construction of the Engineered SD116 Strain by Simultaneously Expressing Pin-O3D and Weakening FAS Expression

**Figure 3:**
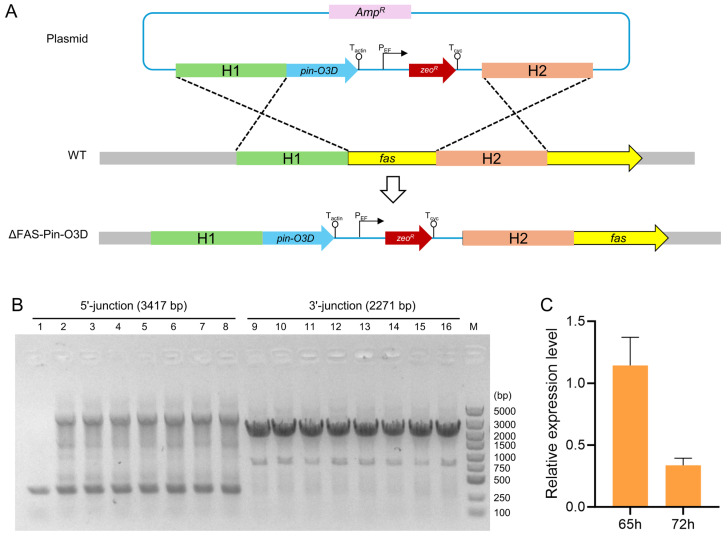
Construction and validation of strain ΔFAS-Pin-O3D. (**A**) Schematic diagram of the plasmid design for constructing ΔFAS-Pin-O3D. (**B**) Nucleic acid electrophoresis validation of integration of the Pin-O3D-Zeo expression cassette into the genome. Lanes 1–8 show 5′ integration fragments, and lanes 9–15 show 3′ integration fragments, corresponding to transformants 1–8, respectively. (**C**) Relative expression levels of Pin-O3D in the ΔFAS-Pin-O3D strain at 65 h and 72 h compared with the level at 48 h. Values are means ± SD (n = 3 independent replicates).

An integration site was required for heterologous gene expression in *Aurantiochytrium* sp. SD116. Our previous study indicated that two copies of *FAS* exist in SD116, and inactivation of one copy by insertion of a zeocin-resistance cassette increased DHA content without affecting growth or lipid yield [23]. This insertion was achieved via homologous recombination, suggesting that the *FAS* locus is an excellent site for heterologous gene integration. Since our goal was to enhance DHA production, combining Pin-O3D expression with FAS weakening represented an ideal and straightforward strategy. This was implemented by inserting both the *Pin-O3D* gene and a zeocin-resistance expression cassette directly downstream of the endogenous FAS promoter while simultaneously deleting the N-terminal part of FAS (Figure 3A). This design attenuated the FAS pathway by inactivating one *FAS* copy and placed *Pin-O3D* under control of the highly active *FAS* promoter, which is expected to drive strong expression during lipid accumulation. After transformation and screening, we obtained transformants with double crossovers at both homologous arms, as verified by diagnostic PCR and agarose gel electrophoresis (Figure 3B). Transformant no. 7 was subjected to further sequencing, which confirmed correct insertion without unintended mutations; the engineered strain was named ΔFAS-Pin-O3D. To confirm functional transcription of the introduced gene, Quantitative real-time PCR (qRT-PCR) was performed at 48 h, 65 h, and 72 h during lipid accumulation. The results demonstrated stable and detectable *Pin-O3D* transcription in ΔFAS-Pin-O3D (Figure 3C), genetically and transcriptionally validating the engineered strain and indicating that it is suitable for downstream phenotypic analysis.

### 2.4. Performance of the ΔFAS-Pin-O3D Strain in Flask Fermentation

**Figure 4:**
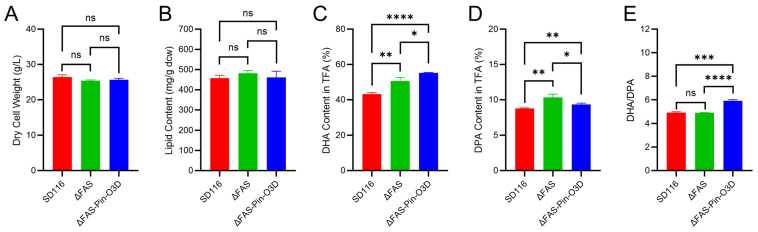
Shake-flask fermentation analysis. Comparison of dry cell weight (**A**), lipid content (**B**), DHA content as percentage of total fatty acids (**C**), DPA content as percentage of total fatty acids (**D**), and DHA/DPA ratio (**E**) of SD116, ΔFAS, and ΔFAS-Pin-O3D. Values are means ± SD (n = 3 independent replicates). Comparisons of means for significant differences were conducted using *t*-tests. ns, not significant; *, *p* < 0.05; **, *p* < 0.01; ***, *p* < 0.001; ****, *p* < 0.0001.

The performance of ΔFAS-Pin-O3D was evaluated under shake-flask fermentation conditions (Figure 4). The previously constructed strain ΔFAS [23] and wild-type SD116 served as controls to assess the individual contributions of FAS weakening and Pin-O3D expression. No significant differences in cell dry weight or lipid content were observed among SD116, ΔFAS, and ΔFAS-Pin-O3D (Figure 4A,B), indicating that inactivation of one *FAS* copy and expression of Pin-O3D did not impose additional metabolic burdens or interfere with overall lipid biosynthesis. Consistent with previous findings [23], ΔFAS showed significantly increased DHA and DPA contents (50.6 ± 2.0% and 10.3 ± 0.4% of total fatty acids, respectively) compared with SD116 (43.2 ± 0.8% and 8.8 ± 0.1%, respectively) (Figure 4C,D), while the DHA/DPA ratio remained similar between ΔFAS and SD116 (Figure 4E). Compared with ΔFAS, ΔFAS-Pin-O3D exhibited a further 9.0% increase in DHA content, reaching 55.2 ± 0.2% of total fatty acids (Figure 4C), accompanied by a 9.7% reduction in DPA content to 9.3 ± 0.2% (Figure 4D). Consequently, the DHA/DPA ratio increased by 20.9%, from 4.89 ± 0.02 in ΔFAS to 5.91 ± 0.10 in ΔFAS-Pin-O3D (Figure 4E). These results demonstrate that Pin-O3D expression effectively promotes DPA-to-DHA conversion in *Aurantiochytrium*, further increasing DHA content on top of weakening FAS activity.

### 2.5. Performance of the ΔFAS-Pin-O3D Strain in Fed-Batch Fermentation

**Figure 5:**
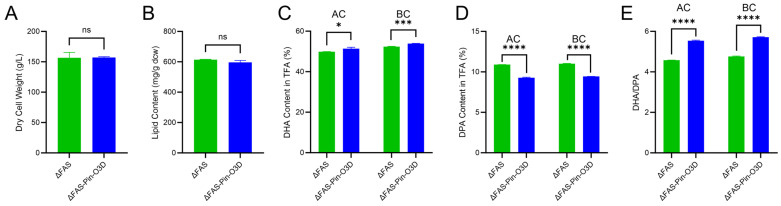
Fed-batch fermentation analysis. Comparison of dry cell weight (**A**) and lipid content (**B**) of ΔFAS and ΔFAS-Pin-O3D. DHA content as percentage of total fatty acids (**C**), DPA content as percentage of total fatty acids (**D**), and DHA/DPA ratio (**E**) of ΔFAS and ΔFAS-Pin-O3D were compared using the acid-catalyzed methyl esterification method (AC) and the base-catalyzed methyl esterification method (BC). Values are means ± SD (n = 3 independent replicates). Comparisons of means for significant differences were conducted using *t*-tests. ns, not significant; *, *p* < 0.05; ***, *p* < 0.001; ****, *p* < 0.0001.

To further validate the engineering strategy under industrially relevant conditions, fed-batch fermentations were performed in a 5-L bioreactor using ΔFAS and ΔFAS-Pin-O3D. The total fatty acid compositions are presented in Table 1. Throughout fermentation, both strains exhibited comparable growth kinetics and lipid accumulation trends. No significant differences in final biomass or total lipid yield were observed between ΔFAS-Pin-O3D and ΔFAS (Figure 5A,B), indicating that Pin-O3D expression remained metabolically compatible with large-scale cultivation. Notably, consistent with shake-flask results, introduction of Pin-O3D substantially reshaped the LC-PUFA profile in the bioreactor. Based on the conventional acid-catalyzed methyl esterification method (H_2_SO_4_–methanol), ΔFAS-Pin-O3D exhibited a 5.1% increase in DHA content and a 13.8% reduction in DPA level compared to ΔFAS (Figure 5C,D). As a result, the DHA/DPA ratio increased by 22.4%, from 4.60 ± 0.06 in ΔFAS to 5.63 ± 0.08 in ΔFAS-Pin-O3D (Figure 5E). To exclude potential effects of high temperature on polyunsaturated fatty acids during methyl esterification, an independent analysis was performed using a mild base-catalyzed method (KOH–methanol). The results showed a slight increase for both DHA and DPA contents compared to the results of the acid-catalyzed method, while a highly consistent trend was observed: compared to ΔFAS, ΔFAS-Pin-O3D displayed a 2.9% increase in DHA content and a 14.1% decrease in DPA level, leading to a 20.15% increase in the DHA/DPA ratio (from 4.76 ± 0.02 to 5.71 ± 0.01). These results confirmed that Pin-O3D-mediated DPA-to-DHA conversion remained effective and robust under fed-batch conditions. Collectively, these results highlight the scalability of the Pin-O3D-based engineering strategy for improving DHA enrichment in *Aurantiochytrium*.

The physiological significance of DPA in *Aurantiochytrium* is currently unclear. Therefore, it is impossible to predict whether alterations in the DPA/DHA ratio will have any effects on the physiological metabolism of *Aurantiochytrium*. Future research could further analyze whether the reduction in DPA varies across different lipid types, particularly among different types of polar lipids, to speculate on the possible physiological roles of DPA.

It is noteworthy that although overexpression of Pin-O3D in *Aurantiochytrium* sp. SD116 significantly increased the DHA/DPA ratio, the DPA conversion ratio was only about 13–14%. While this conversion ratio is substantially higher than previously reported for other O3Ds in *Schizochytrium* [18,20], it remains considerably lower than the ~80% conversion observed in tobacco leaves [22]. Possible reasons include the high DHA/DPA ratio in *Schizochytrium*/*Aurantiochytrium*, which may lead to product inhibition. Additionally, after synthesis by the PUFA synthase and conversion to DHA-CoA, DHA and DPA are ultimately assembled into triacylglycerols (TAGs) and stored in lipid droplets. Our sequence and structure analyses indicate that Pin-O3D is a membrane-bound CoA-type desaturase, suggesting it can only utilize acyl-CoA as substrate. Given the high efficiency of TAG assembly in these organisms, the concentration of DPA in the acyl-CoA pool available for Pin-O3D may be low, limiting conversion efficiency. Future research could seek enzymes with higher conversion efficiency or fuse O3D with TAG synthesis-related enzymes to further enhance conversion.

## 3. Materials and Methods

### 3.1. Strains and Culture Conditions

*Aurantiochytrium* sp. SD116 was previously isolated and preserved in our laboratory [9]. *Escherichia coli* BL21(AI) was purchased from Shanghai Angyu Biotechnology Co., Ltd. (Shanghai, China). *Aurantiochytrium* sp. SD116 was grown at 25 °C in 30 mL of GYS medium (10 g/L glucose, 20 g/L yeast extract, 15 g/L sea salt) and then transferred to 50 mL of GYSF medium (60 g/L glucose, 20 g/L yeast extract, 15 g/L sea salt) for fermentation. *E. coli* BL21(AI) was cultured in LB medium.

### 3.2. Plasmid Construction

The codon-optimized coding sequence of Pin-O3D was synthesized by Sangon Biotech (Shanghai) Co., Ltd. (Shanghai, China). The *E. coli* system for expressing the PUFA synthase was established as previously described [26]. Expression of ORF-A, ORF-B, ORF-C, and PPTase was carried out using pET-Duet and pACYA-Duet vectors. To express Pin-O3D in *E. coli*, an expression cassette containing a ribosome binding site was inserted after the ORF-B coding region in the above system.

To construct the pWKZ-ΔFAS-Pin-O3D-Zeo plasmid, the *Pin-O3D* gene was sequentially fused via overlap extension PCR with the zeocin-resistance gene (*zeo*) expression cassette and homologous fragments corresponding to the promoter and coding regions of the *FAS* gene, following a similar procedure as for pWKZ-FAS [23]. The fused fragment was then inserted into the pGZC-1 vector [23,27], yielding the Pin-O3D expression plasmid pWKZ-ΔFAS-Pin-O3D-Zeo. All primers used in this study are listed in Table 2.

### 3.3. Induction and Fermentation of E. coli

*E. coli* BL21(AI) transformants containing both the PUFA synthase and Pin-O3D were inoculated into LB broth and cultivated overnight at 37 °C with shaking at 200 rpm. Ten milliliters of the bacterial culture was transferred into 1 L of LB broth and grown at 37 °C with agitation until the logarithmic phase (OD_600_ ≈ 0.6). Arabinose (0.4 g/L) and IPTG (0.6 mM) were added to induce protein expression. The temperature was then reduced to 22 °C, and the culture was incubated for 36 h with shaking at 120 rpm. Cells were harvested by centrifugation, and total lipids were extracted for transesterification and fatty acid analysis.

### 3.4. Transformation of Aurantiochytrium sp. SD116

Electrocompetent cells of *Aurantiochytrium* sp. SD116 were prepared according to a previously reported protocol [27] with slight modifications. Briefly, a single colony was inoculated into GYS medium and cultivated at 25 °C for 12 h. The resulting seed culture was subcultured into fresh GYS medium and incubated overnight until the OD_600_ reached 6–8. Cells were collected by centrifugation at 4 °C and gently washed with 20 mL of ice-cold 1 M sorbitol. The cell pellet was resuspended in buffer A (0.6 M sorbitol, 0.1 M LiAc, 10 mM DTT, pH 7.0) and incubated on ice for 30 min. After centrifugation at 4 °C, the cells were resuspended in 3 mL of buffer B (1 M sorbitol, 10 mM K_2_HPO_4_, 5 mM MgCl_2_, pH 7.4). The resulting competent cells were kept on ice and used immediately for electroporation.

For transformation, 90 μL of competent cells were mixed with 2–3 μg of plasmid DNA in a pre-chilled 0.2 cm electroporation cuvette. Electroporation was performed using a custom-built electroporation device (MT01-3KV, QIBEBT-CAS, Qingdao, China) under the following conditions: 1.2 kV, 1000 Hz, 10% pulse duration/interval, 50 square electric pulses. After pulsing, cells were supplemented with GSY medium and incubated at 25 °C for 12 h. Subsequently, the recovered cells were plated onto selective agar medium containing 30 μg/mL zeocin and incubated for 3–5 days at 25 °C. Transformants were inoculated into GSY medium containing 10 μg/mL zeocin and grown for 2 days. Genomic DNA was extracted and used for PCR verification.

### 3.5. qRT-PCR Analysis

Total RNA was extracted from 2 mL of *Aurantiochytrium* sp. SD116 cells harvested at the exponential phase using the FastPure Cell/Tissue Total RNA Isolation Kit (Vazyme, Beijing, China) according to the manufacturer’s instructions. First-strand cDNA was synthesized from purified RNA using the HiScript III 1st Strand cDNA Synthesis Kit (Vazyme, China). qRT-PCR was performed with the primers RT-Pin-F (CTTCCCTACCCTCACCGAGA) and RT-Pin-R (GTGACCCACGGTGAAAAAGC) using ChamQ Universal SYBR qPCR Master Mix (Vazyme, China) and a real-time PCR system. Gene expression levels were normalized against the housekeeping gene *actin*, and relative transcript abundance was calculated using the 2^−ΔΔCt^ method.

### 3.6. Biomass, Lipid, and Fatty Acid Analysis

Biomass was quantified as dry cell weight. Briefly, 2 mL of culture broth was harvested, and the cell pellet was subjected to vacuum freeze-drying at −55 °C overnight. The dried biomass was then weighed gravimetrically.

For shake-flask cultures, total lipids were extracted using a chloroform-methanol solvent system (2:1, *v*/*v*) after acid pretreatment with hydrochloric acid. The organic phase containing lipids was collected and transferred into pre-weighed glass vials. After solvent removal under vacuum at 50 °C, total lipid content was determined gravimetrically. For fed-batch fermentation samples, total lipids were extracted using an enzymatic cell disruption method. Briefly, cells were treated with alkaline protease to facilitate cell lysis, followed by extraction with a hexane–ethanol solvent system. The organic phase was recovered, and solvents were removed by rotary evaporation. An aliquot of the extracted lipids was then dried at 85 °C to constant weight, and total lipid content was calculated gravimetrically.

For fatty acid analysis, two independent methyl esterification methods were applied. In the acid-catalyzed method [28], the extracted lipids were dissolved in 1 mL of chloroform and converted to fatty acid methyl esters (FAMEs) by transesterification with 2% (*v*/*v*) sulfuric acid in methanol at 85 °C for 2.5 h. In the base-catalyzed method, 50 mg of lipids were dissolved in 2 mL of n-hexane and subjected to transesterification with 0.4 mol/L KOH in methanol at 35 °C for 30 min. The resulting FAMEs from both methods were filtered through a 0.22 μm organic membrane filter and transferred into GC vials for subsequent analysis. Base-catalyzed transesterification can be affected by free fatty acids [29]; however, this is not a concern in the present study, as the fatty acids in *Aurantiochytrium* are mainly stored as triacylglycerols in lipid droplets.

FAMEs were extracted with n-hexane and analyzed using an Agilent 7890B gas chromatograph (Agilent Technologies, Santa Clara, CA, USA) equipped with a flame ionization detector. Separation was performed on an HP-INNOWAX capillary column (30 m × 0.25 mm i.d., 0.25 μm film thickness). The injector temperature was set at 250 °C, and samples were introduced in split mode with a split ratio of 9:1. The oven temperature program was as follows: initial temperature of 60 °C; ramped to 180 °C at 15 °C/min; increased to 230 °C at 3 °C/min; followed by a further increase to 250 °C at 10 °C/min and held for 5 min. The detector temperature was maintained at 280 °C. Hydrogen, air, and nitrogen (make-up gas) flow rates were set at 30, 400, and 30 mL/min, respectively. High-purity nitrogen (≥99.999%) was used as the carrier gas at a constant flow rate of 1.2 mL/min. The injection volume was 2.0 μL. Identification of individual fatty acids was based on retention times by comparison with authentic standards and our previous gas chromatography-mass spectrometry analysis [26] in which peak identities were confirmed using the NIST mass spectral library. The content of each fatty acid was quantified based on the GC peak area and expressed as a percentage of total fatty acids.

### 3.7. Fed-Batch Fermentation

Fed-batch fermentation was carried out in a 5-L bioreactor following a previously established procedure [11] with minor modifications. The initial fermentation medium contained 120 g/L glucose, 10 g/L yeast extract, 5 g/L tryptone, 1 g/L MgSO_4_, 5 g/L KH_2_PO_4_, and 15 g/L artificial seawater, with an initial working volume of 2.5 L. Prior to inoculation, 0.75 mL of antifoam agent was added to suppress foam formation. Fermentation was performed at 25 °C with an aeration rate of 2 vvm and an agitation speed of 600 rpm for 144 h. During cultivation, an 80% (*w*/*v*) glucose solution was supplied as the feeding substrate.

### 3.8. Statistical Analysis

All quantitative experiments were performed with three independent replicates. Results in Figure 3C, Figure 4 and Figure 5, and Table 1 are reported as the mean ± standard deviation of the mean. Comparisons of means for significant differences were conducted using *t*-tests. Significant statistical differences are indicated by asterisks: *, *p* < 0.05; **, *p* < 0.01; ***, *p* < 0.001; ****, *p* < 0.0001.

## 4. Conclusions

In this study, we identified and functionally validated Pin-O3D as a membrane-bound ω-3 desaturase that efficiently converts DPA to DHA in *Aurantiochytrium* sp. SD116. Structural analysis revealed conserved catalytic motifs and transmembrane features consistent with its predicted desaturase activity. Heterologous reconstruction of the PKS pathway in *E. coli* demonstrated that Pin-O3D expression markedly shifted the DPA/DHA ratio, providing direct evidence of DPA-to-DHA conversion. Genomic integration of *Pin-O3D* into the *FAS* locus of SD116 enabled coordinated attenuation of the competing FAS pathway and DPA-to-DHA conversion during lipid accumulation. The resulting strain showed increased DHA content and DHA/DPA ratio without affecting growth or total lipid production, and this improvement was maintained under fed-batch fermentation. Together, this study establishes an effective DPA conversion strategy and provides a rational metabolic engineering framework for improving DHA purity in industrial thraustochytrid platforms.

## Figures and Tables

**Table 1:** Fatty acid compositions as percentages of total lipids in ΔFAS and ΔFAS-Pin-O3D.

Fatty Acid	ΔFAS (%)	ΔFAS-Pin-O3D (%)
Acid-catalyzed methylation (H_2_SO_4_–methanol)		
C14:0	1.49 ± 0.01	1.64 ± 0.04
C16:0	33.46 ± 0.04	33.16 ± 0.75
C17:0	0.35 ± 0.01	0.39 ± 0.01
C18:0	1.11 ± 0.01	1.11 ± 0.03
C22:5 (ω-6)	10.90 ± 0.01	9.28 ± 0.07
C22:6 (ω-3)	49.87 ± 0.06	51.39 ± 0.68
Base-catalyzed methylation (KOH–methanol)		
C14:0	1.33 ± 0.01	1.45 ± 0.01
C16:0	30.50 ± 0.12	30.23 ± 0.12
C17:0	0.33 ± 0.01	0.36 ± 0.01
C18:0	1.02 ± 0.01	1.11 ± 0.15
C22:5 (ω-6)	11.01 ± 0.03	9.43 ± 0.02
C22:6 (ω-3)	52.35 ± 0.15	53.86 ± 0.11

**Table 2:** Primers used in this study.

Primer	Sequence
FASH1-F	AATTCGGGTCTTCTTCAAAGCCACAGCGGGTTTTTACTC
FASH1-R	CTTGGTGGCCATGGTTATGGTTTTTCAACAATGAAG
PinO3D-F	ATTGTTGAAAAACCATAACCATGGCCACCAAGGAGGCC
PinO3D-R	GGGCATTCCATCACTCCAATTTAAGTGGACTTGGTCTTGGCG
Tactin-F	CTTAAATTGGAGTGATGGAATGCCCTCTCCGTGTGGTG
Tactin-R	GTGGATTGCATGCGTGACCTTCCGCGTGTCAG
pEF-F	GTCACGCATGCAATCCACCGTCCAACAAC
CYC1-R	GTTCCTGGCCAAAGGCTGAGTTGCAAATTAAAGCCTTCGAGCG
FASH2-F	CTCAGCCTTTGGCCAGGAACTTGCG
FASH2-R	GAATTAGCCATGGTCGAATGTTGCCCATAATATCAGCGATG
Vector-F	CAACATTCGACCATGGCTAATTCGTCGACACGTAG
Vector-R	AAGCAGGGTTATGCAGCGGAAAAATTCGGGTCTTCTTCAAAGC

## Data Availability

Data is contained within the article.

## Abbreviations

The following abbreviations are used in this manuscript:

ARA	arachidonic acid
DHA	docosahexaenoic acid
DPA	docosapentaenoic acid
EPA	eicosapentaenoic acid
FAME	fatty acid methyl ester
FAS	fatty acid synthase
GC	gas chromatography
LC-PUFA	long-chain polyunsaturated fatty acid
O3D	ω-3 desaturase
Pin-O3D	ω-3 desaturase from *Phytophthora infestans*
PKS	polyketide synthase
PPTase	phosphopantetheinyl transferase
PUFA	polyunsaturated fatty acid
qRT-PCR	quantitative real-time PCR
SCD1	stearoyl-CoA desaturase 1
TAG	triacylglycerol
